# Dual-Factor Mental Health from Childhood to Early Adolescence and Associated Factors: A Latent Transition Analysis

**DOI:** 10.1007/s10964-021-01550-9

**Published:** 2021-12-17

**Authors:** Kimberly J. Petersen, Neil Humphrey, Pamela Qualter

**Affiliations:** Manchester Institute of Education, The University of Manchester, Oxford Road, Manchester, M13 9PL UK

**Keywords:** Dual-factor, Mental health, Social support, Latent transition analysis, Longitudinal, Early adolescence

## Abstract

The dual-factor model of mental health indicates the importance of simultaneously assessing symptoms and subjective wellbeing, but there is limited understanding of how dual-factor mental health changes during the transition from childhood to early adolescence and factors associated with change. The current study investigated dual-factor mental health over a 2-year period from when children were 8–9 years old to 10–11 years old (*N* = 2402; 48% female), using latent transition analysis. Further analyses determined whether sex and peer support were associated with initial mental health status or specific transitions during this period. Following class enumeration procedures, a 5-class model was selected at both timepoints. Classes were: (1) *complete mental health*, (2) *vulnerable*, (3) *emotional symptoms but content*, (4) *conduct problems but content*, and (5) *troubled*. Half of the sample changed mental health status during the study period. Sex and peer support were associated with specific mental health statuses and subsequent transitions. The findings have implications for mental health screening practice and identifying those in need of targeted interventions.

## Introduction

Major physical, psychological, and social changes take place as children move into early adolescence, and those can negatively impact mental health (Blakemore, 2019). To support young people through this important developmental period, it is necessary to understand: (1) how mental health changes during this time, (2) who is susceptible to poor mental health, and (3) which factors maintain or promote positive adaptation. Research consistently indicates an overall increase in mental health symptoms (Patalay & Fitzsimons, 2017) and a decrease in subjective wellbeing (Casas & González-Carrasco, 2019) from childhood to adolescence. However, within the population there is a great deal of heterogeneity (von Eye & Bergman, 2003). Some will have deteriorating mental health; others will show stable or improving mental health. Person-oriented statistical approaches, such as latent variable mixture modeling, have been used to elucidate those different developmental patterns (e.g., Shore et al., 2018), but few studies have taken an approach where symptoms of distress and subjective wellbeing are examined simultaneously. The “dual-factor” model of mental health suggests that psychopathology and wellbeing are important and distinct aspects of mental health (Greenspoon & Saklofske, 2001). Therefore, more traditional approaches, which investigate symptoms alone, provide only a partial view of mental health (Keyes, 2002). A handful of studies have investigated dual-factor mental health longitudinally, focusing exclusively on adolescents (Kelly et al., 2012; McMahan, 2012; Moore et al., 2019b; Xiong et al., 2017; Zhou et al., 2020) or young children (Compton, 2016), but the extent that dual-factor mental health changes during the transition *from* childhood *to* early adolescence is unknown. In addition, factors predicting changes in mental health during this period have not been explored. The current study addresses those gaps by taking a person-oriented approach to investigate dual-factor mental health from childhood (age 8–9 years) to early adolescence (age 10–11 years), and investigating whether sex and perceived peer support are associated with initial mental health status or specific transitions during the study period.

### The Dual-Factor Model of Mental Health

The dual-factor model emphasizes the importance of measuring both psychopathology (i.e., mental health symptoms) and subjective wellbeing (i.e., overall positive affect and life satisfaction; Diener, 2000) for the comprehensive assessment of mental health (Greenspoon & Saklofske, 2001). While traditional disease-based models focus on a single mental health continuum from symptomatic to asymptomatic, the dual-factor model posits that psychopathology and subjective wellbeing are distinct factors (Iasiello et al., 2020). Logically, a minimum of four mental health subgroups can be derived from this model: (a) low symptoms and high subjective wellbeing (*complete mental health*); (b) high symptoms and low subjective wellbeing (*troubled*); (c) low symptoms and low wellbeing (*vulnerable*); and (d) high symptoms and high wellbeing (*symptomatic but content*). Studies have consistently identified those subgroups among children and adolescents and have further indicated that they are associated with distinct determinants and outcomes, demonstrating the need to assess mental distress and wellbeing simultaneously in order to identify those important subgroups (Smith et al., 2020). A further strength of the dual-factor approach is that it captures differences in mental health even when individuals are asymptomatic (Wang et al., 2011). Studies that focus on symptoms alone capture mental health development in a small, symptomatic subset of the population. By contrast, studies that incorporate data on symptoms *and* wellbeing provide greater insights on mental health in the general population (given that the majority of the population are asymptomatic, variability in subjective wellbeing is important; Black et al., 2021). Furthermore, dual-factor research can inform early identification of those who may later become symptomatic (e.g., the *vulnerable* class noted above), while also helping to distinguish between symptomatic cases that are struggling (e.g., those who are *symptomatic but content*) and those that are languishing (e.g., the *troubled* class). Such insights have important implications for the allocation of scarce resources to mental health in schools and other settings. Finally, empirical support for the dual-factor approach has been found in a range of study designs (e.g., cross-sectional, longitudinal, and intervention studies), in clinical and nonclinical samples, in Western and non-Western populations, and across the lifespan (Iasiello et al., 2020). Therefore, to better understand mental health development from childhood to early adolescence, changes in patterns of mental distress *and* subjective wellbeing must be considered.

### Methods of Investigating Dual-Factor Mental Health

To investigate dual-factor mental health, researchers have typically identified the subgroups noted above using sample or norm-based cut-off points. Recently, approaches such as latent class analysis (LCA) and latent profile analysis (LPA; Berlin et al., 2013) have been used to identify subgroups of children and adolescents with similar patterns of dual-factor mental health (e.g., Petersen et al., 2020). LCA and LPA are model-based clustering techniques that identify underlying subgroups based on different indicator variables (Collins & Lanza, 2010). Those methods have several advantages over cut-point techniques. First, they identify subgroups that are internally homogenous and externally heterogenous (Berlin et al., 2013). Second, classification is probabilistic, thus accounting for measurement error (Distefano & Kamphaus, 2006). Third, because LCA and LPA are data-driven, they do not presuppose the nature of the subgroups that will be identified, although theory can be used to guide class selection (Collins & Lanza, 2010). Indeed, previous LCA and LPA studies have identified dual-factor mental health subgroups beyond the four derived using cut-point methods, for example “symptomatic but content” subgroups which differ by symptom dimension (i.e., internalizing/externalizing; Petersen et al., 2020), or a subgroup with moderate symptoms and moderate wellbeing (Moore et al., 2019a). In sum, LCA and LPA are more advanced, empirically based, and flexible methods for investigating dual-factor mental health than traditional approaches.

### Investigating Dual-Factor Mental Health Longitudinally

Few studies have investigated dual-factor mental health *longitudinally*. Those that have, mainly used cut-point scores to identify the four logically derived dual-factor subgroups and examined the extent to which participants changed groups over time. Those studies suggested that dual-factor mental health among children age 5–7 years (Compton, 2016), and adolescents age 11–13 years (Kelly et al., 2012), 14-17 years (McMahan, 2012) and 12–18 years (Xiong et al., 2017), is relatively stable over periods ranging from five-months to one-year, particularly for those with *complete mental health*. Across those studies, 80–86% of each sample remained in the *complete mental health* group. However, other groups were less stable, with 24-44% remaining in the *vulnerable*, 17–44% remaining in the *symptomatic but content*, and 35–61% remaining in the *troubled* group over varying time periods. In addition, a smaller number of studies employed longitudinal extensions of LCA and LPA, called latent transition analysis (LTA). Those studies identified slightly different dual-factor subgroups among adolescent samples. For example, one study identified *complete mental health, moderately mentally healthy, symptomatic but content*, and *troubled* subgroups among 13–18 year olds (Moore et al., 2019b), and another identified *flourishing/complete mental health*, *vulnerable*, and *troubled* subgroups among 10–15 year olds (Zhou et al., 2020). Results cannot be directly compared due to differences in the sample used, mental health indicators, and time lags, but *complete mental health* tended to be the most stable group, and results highlighted the extent to which others followed different developmental pathways.

Longitudinal dual-factor studies provide a rich picture of mental health development. However, none of the aforementioned studies investigated dual-factor mental health development during the period from childhood to early adolescence, yet this is crucial given that this unique developmental phase is marked by cognitive, neurological, social, and hormonal change (Blakemore, 2019) and contextual developments. For example, in England, the beginning of adolescence coincides with the final year of primary school and involves national high stakes tests (Standards and Testing Agency, 2019), meaning that young adolescents are introduced to new responsibilities and stressors. Thus, it is important to understand how mental health changes during that time. Accordingly, the current longitudinal study was designed to enable a comparison of dual-factor mental health statuses at age 8–9 and 10–11 years, at the group level (i.e., do new status patterns emerge and/or do some disappear? What is the prevalence of the different statuses at each point in time?), *and* to test the temporal stability of mental health statuses within individuals (i.e., how stable is membership of a particular status?). While such an exploration has not been conducted before, and therefore our study is exploratory in many ways, based on previous work and the unique developmental changes in this phase of life, we might expect that complete mental health would be the largest and most stable profile, however a substantial proportion of children will show deteriorating mental health during that period (e.g., moving from *complete mental health* to a symptomatic status) and it will be important to identify which young people are more likely to make those changes.

### Factors Associated with Dual-Factor Mental Health

Sex is a key demographic characteristic which has been studied in relation to mental health. However, it is not clear whether males and females are more likely to have particular profiles of dual-factor mental health, as studies have found mixed results with some identifying sex differences (e.g., Moore, 2017) and others failing to do so (e.g., Kelly et al., 2012). However, if dual-factor profiles were also distinguished by symptom type, sex differences might be expected in profile membership and stability of profiles from childhood to early adolescence, given that males are more likely to have conduct problems and females more likely to have emotional problems during this period (Merikangas et al., 2009). In addition, given that adolescence is a turbulent period in development, where youth have to manage stressful hormonal, social, identity, and academic challenges, it could be expected that those who move out of complete mental health transition will migrate to profiles characterized by conduct problems (males) or emotional problems (females). Thus, examination of sex difference is important to be able to identify those at risk of specific patterns of deteriorating mental health.

Relations between psychosocial factors, such as peer support, and dual-factor mental health among young people have also been investigated. For example, studies suggest that those with *complete mental health* typically have higher peer support than those in the *vulnerable* group (Petersen et al., 2020; Smith et al., 2020) and those in the *troubled* group typically have lower peer support than those in the *symptomatic but content* group (Smith et al., 2020; Suldo & Shaffer, 2008). This demonstrates a link between high social support from peers and membership of the complete mental health subgroup, and a link between low support from peers and membership of suboptimal mental health subgroups. Social support may also be most strongly associated with the wellbeing aspect of mental health as both *vulnerable* and *troubled* groups are characterized by low wellbeing. However, few studies have investigated whether peer support predicts changes in dual-factor mental health over time. An exception found that peer relationship stress was associated with some patterns of deteriorating dual-factor mental health among Chinese adolescents (Zhou et al., 2020). Further research is needed to establish whether similar relations are found among younger individuals as associations between peer support and mental health are typically stronger with older adolescents (Rueger et al., 2016).

## Current Study

To improve understanding of how dual-factor mental health develops from childhood to early adolescence, the current study used LCA to investigate which dual-factor mental health classes were identified among 8–9 year olds and, two years later, when they were age 10–11 years. Further, LTA was used to investigate the extent to which children transitioned from one mental health class to another as they entered early adolescence, and the nature of transitions during that period. Those analyses were necessarily exploratory because there are discrepancies in the literature regarding the classes that are identified when multiple symptoms and wellbeing are used as mental health indicators, and no study has yet investigated dual-factor mental health transitions with this age group. However, based on extant literature, some general predictions were made, for example it was predicted that a large *complete mental health* subgroup would be identified (i.e., low symptoms and high wellbeing) and that this would be the most stable class, since most children and adolescents in the general population experience good mental health. A secondary aim was to investigate factors associated with changes in dual-factor mental health during this important developmental period. Therefore, the current study also investigated whether sex or perceived peer support were associated with initial mental health statuses (at age 8–9 years) or subsequent mental health transitions as they approached early adolescence. It was expected that sex differences would be seen when mental health classes were distinguished by symptom type, for example males would be more likely to have (or change to) a status that is distinguished by high externalizing symptoms, whereas females would be more likely to have (or move to) a status distinguished by high internalizing symptoms. Further, because high social support from peers is a promotive factor for mental health and low social support from peers is a risk factor, it was predicted that high perceived peer support would be associated with having a mental health status that represented good mental health (e.g., *complete mental health*) or positive changes in mental health (e.g., from a status indicating high symptoms and/or low wellbeing to *complete mental health*). Conversely, low perceived peer support would be associated with statuses indicating suboptimal mental health, and/or deterioration in mental health as children entered early adolescence.

## Methods

### Participants and Procedure

This study used data collected in the follow-up phase of a randomized controlled trial (RCT) of the Good Behavior Game (further details of the original study can be found in Humphrey et al., 2018). The current study used data from 2402 pupils for whom there was mental health data when they were in Year 4 (age 8–9 years, summer term 2017) and Year 6 (age 10–11 years, summer term 2019) of the English education system. Participants attended 76^1^ primary schools in England that participated in the RCT. Participant sociodemographic information is presented in Table 1 (average responses on mental health and social support measures are presented in Online Resource 1).

**Table 1:** Sociodemographic characteristics of the study sample

	frequency	%
Male	1257	52
FSM eligible	567	24
With SEND	439	18
*Ethnicity*		
White	1554	65
Asian	508	21
Black	109	5
Chinese	8	<1
Mixed	134	6
Any other ethnic group	70	3
Unclassified	11	1

*Note*. FSM free school meals, SEND special educational needs and disabilities.

### Measures

#### Mental health difficulties

Mental health difficulties were assessed using individual items from the emotional symptoms and conduct problems subscales of the UK teacher-report version of the Strengths and Difficulties Questionnaire (SDQ; Goodman, 1997). Conduct problems and emotional symptoms were chosen because they related to the two key dimensions of psychopathology identified among children (Goodman et al., 2010). Individual items were used rather than subscale scores so that subgroups could differ in terms of one or more specific symptoms. Conduct problem items were: (1) *Often has temper tantrums or hot tempers*, (2) *Generally obedient, usually does what adults request* (reverse scored), (3) *Often fights with other children or bullies them*, and (4) *Often lies or cheats*^2^. The emotional symptom items were: (1) *Often complains of headaches, stomach-aches or sickness*, (2) *Many worries, often seems worried*, (3*) Often unhappy, down hearted or tearful*, (4) *Nervous or clingy in new situations, easily loses confidence*, and (5) *Many fears, easily scared*. The SDQ asks teachers to rate whether the statements are “not true”, “somewhat true”, or “certainly true”. For the LCA, responses were dichotomized so that 0 = “not true”, and 1 = “somewhat” or “certainly true”. The distribution of scores and the meaning of those responses suggested dichotomization in this way was appropriate. The majority of responses were “never” (i.e., showing no symptoms). A smaller number indicated endorsement of symptoms as either “somewhat” or “certainly true”. Similar studies have followed that procedure (Petersen et al., 2020; Wadsworth et al., 2001). Although some information about symptom severity was lost, dichotomization reduced model complexity, which was necessary for the model to be able to converge on a final solution in Mplus.

#### Subjective wellbeing

Subjective wellbeing was measured using the positively worded items from the psychological wellbeing scale from the self-report version of the KIDSCREEN-27, which has been validated with this age group (Ravens-Sieberer et al., 2007). Those items were chosen because they measured psychological wellbeing rather than symptoms and thereby assessed an additional aspect of mental health. Again, items were used instead of subscale scores, to allow mental health statuses to differ by one or more aspect of wellbeing. The items included in the LCA were: “In the last week… (1) *has your life been enjoyable?* (2) *have you been in a good mood?* (3) *have you had fun?*, and (4) *have you been happy with the way you are?”*. Children responded on a 5-point Likert scale from, 1 = “not at all” or “never”, to 5 = “extremely” or “always”. Treating responses as categorical resulted in a complex model, leading to model estimation difficulties. Unlike the SDQ items, the distribution of responses did not indicate a clear point for dichotomization and there was no precedent for dichotomizing those scores. Further, the items were rated on a 5-point Likert scale, which, arguably, can be treated as continuous data (Rhemtulla et al., 2012). Therefore, those item responses were treated as continuous.

#### Perceived peer support

Perceived support from peers was assessed using the “social and peer support” subscale from the KIDSCREEN-27 survey (Ravens-Sieberer et al., 2007) which participants completed when in Year 4 (age 8–9 years). This is a self-report scale. The subscale consisted of four items with responses on a 5-point Likert scale (1 = never, 2 = seldom, 3 = quite often, 4 = very often, 5 = always). Items were: “Thinking about last week… (1) *have you spent time with your friends?* (2) *have you had fun with your friends?* (3) *have you and your friends helped each other?* (4) *have you been able to rely on your friends?”*. Rasch scores for the scale were translated into T-values with M = 50 and SD = 10, to aid interpretation. Rasch scores could be calculated if a single item score was missing (Ravens-Sieberer, 2006). Studies show that the subscale has good internal reliability (Cronbach’s alpha for both scales = 0.81; Robitail et al., 2007), criterion validity (correlations with scales from the KIDSCREEN-52 item questionnaire of *r* = 0.96; Ravens-Sieberer et al., 2007), and good test-retest reliability over a 2-week period (ICC = 0.61 and 0.74 respectively; Ravens-Sieberer et al., 2007).

#### Sex

Sex was included as a covariate. This information was obtained from the National Pupil Database (NPD; Jay et al., 2019). The NPD is a database governed by the Department for Education in England which contains data on individuals age 2–21 years in state funded education and higher education in England. These data span sociodemographic (e.g., sex, age, ethnicity, special educational needs) and educational (e.g., attainment, attendance, exclusions) domains.^3^

### Data Analysis Plan

LCA and LTA were used to classify participants into mental health classes/statuses based on different indicators of emotional symptoms, conduct problems, and subjective wellbeing, and to investigate transitions from one mental health status to another as children entered early adolescence. Covariates were added to the LTA model to investigate whether sex or perceived peer support were associated with initial mental health status at age 8–9 years or mental health transitions. The arm of the original intervention trial, from which the data was taken, was also included as a covariate to control for any potential intervention effect on mental health status or mental health transitions^4^.

Analyses were conducted in Mplus v8.3, using maximum likelihood estimation and an expectation–maximization (EM) algorithm (Muthén & Muthén, 1998-2017). The complex structure of the data, due to clustering by school, was accounted for using a sandwich estimator (i.e., using the command “type = complex”). Analysis was conducted in the following stages:

#### Cross-sectional LCA at both time-points

Exploratory LCA was carried out at both time points. Models with an increasing number of classes from 1 to 10 were specified, unless convergence issues were encountered. The best fitting model was selected using a range of fit-statistics and substantive considerations. Lower values for information criteria such as Bayesian Information Criteria (BIC), sample size adjusted BIC (ssaBIC), and Akaike Information Criteria (AIC), indicated better model fit. Significant (*p* < 0.05) values on the Lo-Mendell Rubin likelihood ratio test (LMR-LRT) indicated that the k-class model was a significantly better fit than the k-1 class model. Distinctiveness of classes, interpretability, theoretical relevance, parsimony, and smallest class size were also considered (Masyn, 2013).

#### Invariance testing

Longitudinal structural invariance of mental health classes was tested before the final LTA analysis, to ensure that latent classes represented the same constructs over time. Being able to assume structural invariance is preferable to ease interpretation of transition probabilities (Collins & Lanza, 2010). In order to investigate the longitudinal invariance of mental health classes over time, a systematic procedure was followed (i.e., Morin & Litalien, 2017). Similarity was examined in the following order: (1) *configural similarity* (i.e., whether the number of mental health classes were the same at each time point), (2) *structural similarity* (i.e., whether within-class item response probabilities or item means were similar at each time point), (3) *dispersion similarity* (i.e., whether within-class variability for continuous indicators was also the same at each time point), and (4) *distributional similarity* (i.e., whether the prevalence’s of mental health classes were the same at each time point; Morin et al., 2016). Measurement invariance was indicated by lower information criteria values (i.e., BIC, ssaBIC, AIC, and CAIC) for a restricted model compared to the unrestricted model, as that suggested that the restricted model was a better fit when parsimony is accounted for.

#### Three-step LTA with covariates

LTA models are comprised of a measurement model (i.e., latent mental health classes at both time points) and the autoregressive relationship between those classes (Collins & Lanza, 2010). The parameters obtained from LTA are the latent classes (called statuses in LTA), their prevalence at each time point, and transition probabilities. Transition probabilities indicated the probability that individuals with each mental health status at Time 1 (age 8–9 years) would transition to each of the other mental health status at Time 2 (age 10–11 years).

Rather than specifying the measurement model and autoregressive pathways in a single step, a bias-adjusted three-step method was used (Asparouhov & Muthén, 2013). This involved: (1) the measurement model being specified without the regression line from each time point, (2) saving the most likely class and classification error for each individual, and (3) regressing mental health classes from Time 2 on mental health class on Time 1. A strength of this method is that the original class structure and the probabilistic nature of class assignment (i.e., classification error) are preserved in the final LTA model. If regression pathways are included in a single step, they can change the class structure, making results difficult to interpret (Vermunt, 2010). In addition, the covariates were added in the final step so that sex and peer support were regressed onto mental health status at age 8–9 years and on transitions (Nylund-Gibson et al., 2014). The intervention trial arm (intervention versus controls) was also included as a covariate to account for any intervention effects. Again, because covariates were added in the third step, they did not change the nature of the latent statuses.

### Missing Data

Missing data on individual mental health items ranged from 6.2% to 8.3%. Missing data on LCA and LTA indicator variables were imputed by default in Mplus using full information maximum likelihood (FIML; Enders, 2013). There were no missing data on sex or trial arm variables, and 6.7% missing on the peer support variable. Cases with missing data on covariates are automatically deleted list-wise in Mplus, which left 2240 cases in the analysis. In order to include all cases, the variance of covariates was specified in the model statement. This allowed missing data on covariates to be imputed using FIML, resulting in use of the whole sample (*N* = 2402).^5^

## Results

### Latent Class Analysis at Each Time Point

Criteria for selecting the best LCA models are presented in Table 2. Information criteria (BIC, AIC and ssaBIC) continued to decrease as the number of classes increased. The LMR-LRT indicated a 4-class model for children aged 8–9 years and a 5-class model when they were age 10–11 years. The 4- and 5-class models were examined further (plots of average item response probabilities and mean scores for each LCA model are presented in Online Resource 2). Both models identified classes that aligned with those of the dual-factor model, i.e., *complete mental health*, *vulnerable*, and two *symptomatic but content groups* that were differentiated by symptom type (conduct problems or emotional symptoms). The key difference between the 4- and 5- class models was that the 5-class model identified an additional subgroup that had moderate symptoms (both emotional symptoms and conduct problems) and low wellbeing. This had characteristics of the *troubled* group that is posited by the dual-factor model. The 5-class model was chosen because it contained all analogous groups indicated by the dual-factor model and classes appeared structurally similar at each time point. The classes in the 5-class model were: (1) a class with very low probability of symptoms and high wellbeing named *complete mental health* (CMH), (2) a class with similarly low probability of symptoms but low wellbeing named *vulnerable* (Vul), (3) a class with high probability of conduct problems, low to moderate emotional symptoms, and high wellbeing named *conduct problems but content* (CPbC), (4) a class with very low conduct problems, high emotional symptoms, and high wellbeing named *emotional symptoms but content* (ESbC), and (5) a class with moderate to high emotional symptoms and conduct problems, and low wellbeing named *troubled* (Trb).

**Table 2:** Information used to evaluate the latent class model

	k	ll	AIC	BIC	ssaBIC	LMR-LRT	A-LMR-LRT	Proportion per class (model-based)	Entropy
T1	1	−22456.58	44947.16	45045.49	44991.48	n/a	n/a	1.00	n/a
	2	−21305.17	42672.34	42851.64	42753.15	<0.001	<0.001	0.33, 0.67	0.75
	3	−20505.80	41101.61	41361.89	41218.91	<0.001	<0.001	0.19, 0.24, 0.57	0.82
	**4**	−**20069.73**	**40257.45**	**40598.71**	**40411.26**	**<0.001**	**<0.001**	**0.12, 0.14, 0.17, 0.57**	**0.85**
	5	−19811.37	39768.74	40190.97	39959.04	0.717	0.718	0.06, 0.11, 0.14, 0.14, 0.54	0.85
	6	best ll not replicated							
T2	1	−21917.41	43868.82	43967.15	43913.14	n/a	n/a	1.00	n/a
	2	−20410.822	40883.64	41062.95	40964.46	<0.001	<0.001	0.34, 0.66	0.79
	3	−19775.942	39641.88	39902.17	39759.19	0.005	0.006	0.15, 0.21, 0.65	0.85
	4	−19248.01	38614.02	38955.28	38767.82	0.001	0.001	0.12, 0.15, 0.16, 0.58	0.84
	**5**	−**18894.263**	**37934.53**	**38356.76**	**38124.83**	**0.001**	**0.001**	**0.08, 0.12, 0.15, 0.24, 0.41**	**0.80**
	6	−18718.75	37611.50	38114.71	37838.29	0.085	0.087	0.03, 0.08, 0.11, 0.15, 0.24, 0.39	0.81
	7	−18566.232	37334.46	37918.65	37597.75	0.113	0.116	0.03, 0.08, 0.10, 0.12, 0.14, 0.16, 0.37	0.81
	8	−18459.354	37148.71	37813.88	37448.50	0.486	0.489	0.03, 0.05, 0.06, 0.10, 0.12, 0.12, 0.17, 0.36	0.81
	9	−18348.00	36953.99	37700.14	37290.28	0.559	0.561	0.02, 0.03, 0.05, 0.06, 0.09, 0.11, 0.12, 0.18, 0.34	0.82
	10	−18272.84	36831.68	37658.8	37204.46	0.507	0.508	0.02, 0.03, 0.04, 0.05, 0.06, 0.07, 0.09, 0.12, 0.18, 0.33	0.82

*Note*. Bolded rows indicate models with the best fit.

*k* number of classes, ll log-likelihood, AIC akaike information criteria, BIC Bayesian information criteria, ssaBIC sample size adjusted BIC, LMRT Lo-Mendell-Rubin likelihood test, A-LMR-LRT adjusted LMR-LRT, T1 time 1 (age 8–9 years), T2 time 2 (age 10–11 years).

### Exploring Longitudinal Measurement Invariance

Configural similarity was indicated as the 5-class model fit the data well at both time points. A model of configural similarity was estimated in which five mental health classes were simultaneously estimated at both time points without the autoregressive pathways of the LTA. A series of restricted models representing structural, dispersion, and distributional similarity were then estimated (see Online Resource 3 for the fit statistics for each model). Compared to the configural model, BIC was lower for the restricted model that indicated structural similarity. This suggested structural measurement invariance because the model that had constrained item-response probabilities and item means to be equal for comparable statuses at both time points was a better fit than the freely estimated model, when parsimony was taken into account. However, ssaBIC, AIC and CAIC indicated that the configural model fit the data best, suggesting that classes were not invariant over time. Examination of the item response probabilities and means for each class at both time points did not suggest differences that was so substantial that classes could be labeled differently. Therefore, retaining the configural model for the LTA (i.e., without constraining item responses to be equal) would make interpretation of transitions problematic because classes labeled the same would represent slightly different constructs (though not so different that they could be considered different mental health subtypes). Hence, it is recommended that structural invariance be assumed when reasonable to do so (Collins & Lanza, 2010). Because there was some evidence of structural invariance (i.e., lower BIC for the model indicating structural similarity) structural similarity was assumed and item response probabilities and means were held equivalent in the subsequent LTA.

### Latent Transition Analysis

#### The final LTA model

**Fig. 1:**
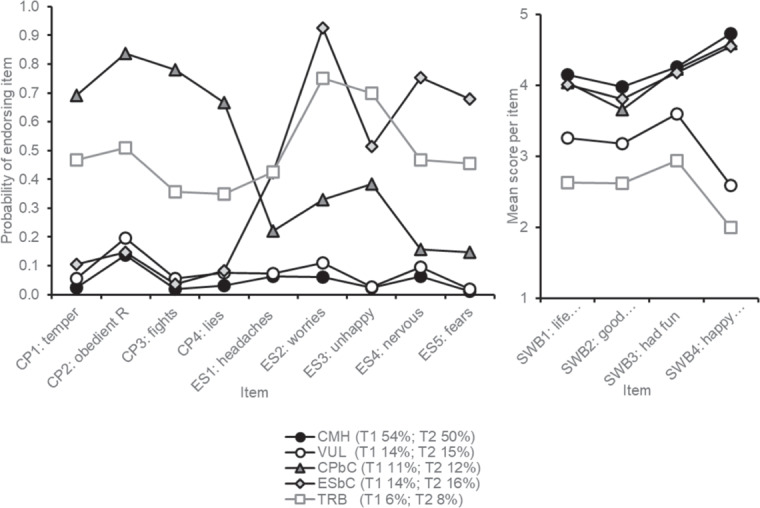
Plot of average reponses for each mental health status in the final latent transition model note. Percentage of children with each mental health status at both time points is provided in the legend. CP conduct problems, ES emotional symptoms, SWB subjective wellbeing, CMH complete mental health status, Vul vulnerable status, CPbC conduct problems but content status, ESbC emotional symptoms but content status, Trb troubled status, T1 time 1 (age 8–9 years), T2 time 2 (age 10–11 years)

The probability of endorsing conduct problem and emotional symptom items, and mean scores on subjective wellbeing items, are shown for each mental health status (see Fig. 1). The prevalence of each mental status at both time points is indicated in the figure legend. The most prevalent class at both timepoints was *complete mental health* (54% at T1; 50% at T2). Prevalence’s are similar over time with a slight reduction of those with CMH status and consequent increase in the proportion of those with a Trb, ESbC, CPbC, and Vul statuses in early adolescence.

#### Prevalence of transition patterns

Prevalence of transition patterns are presented in Table 3. They indicate that the most common transition pattern (representing c.35% of the sample) is to have CMH both in mid-late childhood and early adolescence. In terms of stability, 51% had the same mental health status (whether that be CMH, Vul, ESbC, CPbC or Trb) in early adolescence as they did in mid-late childhood.

**Table 3:** Model-based counts and proportions for each latent transition pattern

Mental health status age 8–9 years	Mental health status age 10–11 years	Model count	Proportion
Complete mental health	**Complete mental health**	**838.76**	**0.35**
	Vulnerable	167.98	0.07
	Emotional symptoms but content	165.93	0.07
	Conduct problems but content	82.86	0.03
	Troubled	48.61	0.02
Vulnerable	Complete mental health	167.09	0.07
	**Vulnerable**	**89.18**	**0.04**
	Emotional symptoms but content	35.75	0.01
	Conduct problems but content	27.35	0.01
	Troubled	24.12	0.01
Emotional symptoms but content	Complete mental health	119.97	0.05
	Vulnerable	53.84	0.02
	**Emotional symptoms but content**	**125.43**	**0.05**
	Conduct problems but content	9.66	<0.01
	Troubled	36.55	0.02
Conduct problems but content	Complete mental health	54.84	0.02
	Vulnerable	24.96	0.01
	Emotional symptoms but content	18.70	0.01
	**Conduct problems but content**	**131.49**	**0.05**
	Troubled	28.91	0.01
Troubled	Complete mental health	27.19	0.01
	Vulnerable	15.83	0.01
	Emotional symptoms but content	28.76	0.01
	Conduct problems but content	29.82	0.01
	**Troubled**	**48.50**	**0.02**

*Note*. Bolded values indicate those that had the same mental health status at both time points.

#### Transition probabilities

Transition probabilities are presented in Table 4. They indicate that the most stable status was CMH; 64% of those with CMH at age 8–9 years had CMH at age 10–11 years. The least stable was the Vul status; only around a quarter of those with Vul status at age 8–9 years retained it at age 10–11 years. Of the symptomatic groups, children with ESbC status had a 51% probability of moving to a non-symptomatic group (Vul or CMH). Whereas, CPbC had a 31% probability, and Trb had a 29% probability, of moving to a non-symptomatic status by early adolescence.

**Table 4:** Latent transition probabilities

		T2 mental health status				
		*CMH*	*Vul*	*ESbC*	*CPbC*	*Trb*
T1 mental health status	CMH	**64%**	13%	13%	6%	4%
	Vul	49%	**26%**	10%	8%	7%
	ESbC	35%	16%	**36%**	3%	11%
	CPbC	21%	10%	7%	**51%**	11%
	Trb	18%	11%	19%	20%	**32%**

*Note*. Bolded values indicate the probability of retaining the same status.

CMH complete mental health, Vul vulnerable, CPbC conduct problems but content, ESbC emotional symptoms but content, Trb troubled, T1 time 1 (age 8–9 years), T2 time 2 (age 10–11 years).

#### Covariate results

Males were more likely than females to have the CPbC status (OR = 2.69, 95% CI [1.84, 3.94]) and less likely to have the ESbC status (OR = 0.71, 95% CI [0.52, 0.98]). Sex was also associated with specific transitions from one mental health status to another. Those who made the transition from CMH to either ESbC or Vul, were *less* likely to be male (ORs = 0.40 and 0.53, 95% CIs [0.25, 0.65], [0.32, 0.87] respectively), and so were those that made the transition from Vul to ESbC (OR = 0.23, 95% CI [0.07,0.73]). However, those making the transition from CMH to CPbC were *more* likely to be male (OR = 2.80, 95% CI [1.36, 5.76]). Other associations between sex and mental health status/transitions were nonsignificant.

Perceived peer support was concurrently associated with mental health status at age 8–9 years. Those with a Vul or Trb mental health status had lower peer support compared to the CMH group (ORs = 0.94 and 0.91, 95% CIs [0.92, 0.96], [0.89, 0.93] respectively). In addition, peer support at age 8–9 years was associated with several transitions. Specifically, those making the transition from CMH to Vul (compared to remaining in CMH) had lower peer support at age 8–9 years (OR = 0.98, 95% CI [0.96, 0.99]). Further, those with ESbC status at 8–9-years-old, who either remained in that class or moved to the Trb class, had significantly lower peer support than those who moved to CMH (ORs = 0.95 and 0.94, 95% CIs [0.91, 0.99], [0.88, 0.99] respectively). Other associations between perceived peer support and mental health status/transitions were nonsignificant.^6^

## Discussion

The dual-factor model of mental health indicates the importance of simultaneously measuring symptoms and subjective wellbeing for a more comprehensive account of mental health, yet little is known about how dual-factor mental health changes during key developmental periods or which factors are associated with change. The primary aim of the present study was to investigate dual-factor mental health development from childhood to early adolescence. A secondary aim was to investigate sex differences and whether perceived social support from peers predicted changes in mental health. LCA and LTA indicated five important mental health statuses at both time points. Those were: *complete mental health*, *vulnerable*, *emotional symptoms but content, conduct problems but content*, and *troubled*. Approximately half the sample changed mental health status during this time. The most prevalent and stable status was *complete mental health*; the *vulnerable* status was the least stable. Changes in dual-factor mental health indicating recovery and deterioration were observed, and sex and levels of perceived support from peers predicted some of those changes. Overall, findings indicated that dual-factor mental health was changeable during this important developmental period, and sex and peer support were linked to specific transitions that indicated deteriorating mental health during that time.

### Comparing Dual-Factor Mental Health Subtypes in Childhood and Early Adolescence

The study investigated and compared dual-factor mental health subtypes in childhood and early adolescence. In childhood, four mental health subtypes were identified. Those were consistent with the dual-factor model, with the addition of symptomatic subgroups differentiated by symptom dimension. The four classes were: (1) *complete mental health*, (2) *vulnerable*, (3) *emotional symptoms but content*, and (4) *conduct problems but content*. The same mental health classes were identified in a previous latent class study that used a subset of the current sample and another sample of children age 8–9-years (Petersen et al., 2020). However, the current study found that, by early adolescence, a 5-class model, which included a new co-morbid *troubled* class, fit the data better. This change may be explained by the tendency for subjective wellbeing to decrease from childhood to adolescence and for symptoms to increase (Casas & González-Carrasco, 2019; Patalay & Fitzsimons, 2017), making the *troubled* group more prominent and easily identifiable. As predicted, the *complete mental health* status was the most prevalent at both time points, which is consistent with previous findings (Compton, 2016; Kelly et al., 2012; McMahan, 2012; Moore et al., 2019b; Xiong et al., 2017). Results from the LTA indicated that the proportion of young people with each mental health status was similar at both time points, albeit with a slightly lower proportion in the *complete mental health* group (54% to 50%), and consequent increase in suboptimal mental health groups by early adolescence. This echoes patterns seen in variable-oriented research, which indicates a trend towards worsening mental health in the overall population (Casas & González-Carrasco, 2019; Patalay & Fitzsimons, 2017). However, small changes in class prevalence’s at each time point concealed a greater degree of change at the individual level.

### Dual-Factor Mental Health Transitions from Childhood to Early Adolescence

The study also investigated the extent to which children transitioned from one dual-factor status to another as they entered adolescence. Latent transition patterns revealed substantial changes in dual-factor mental health over this two-year period; approximately half of the sample moved mental health status during this time. Previous studies reported higher levels of stability in younger and older samples (Compton, 2016; Kelly et al., 2012; McMahan, 2012; Xiong et al., 2017) but examined periods of less than a year, giving little time for change to occur. A recent study investigating dual-factor mental health among 14–18 year olds, over periods of 1-, 2- and 4- years, showed that instability increased over time, and probabilities of transition over a two-year period were similar to those found in the current study (e.g., probabilities of retaining the same mental health status ranged from 0.27 for the *troubled* status to 0.73 for *complete mental health*; Moore et al., 2019b). Thus, findings indicate substantial fluidity in dual-factor mental health over a two-year period from childhood to early adolescence. However, further research using comparable time lags and mental health measurements is needed to establish whether dual-factor mental health stability is lesser or greater during the period from childhood to early adolescence, compared to other age groups.

Consistent with previous research, *complete mental health* was the most stable status (Compton, 2016; Kelly et al., 2012; McMahan, 2012; Moore et al., 2019b; Xiong et al., 2017). In general, children have good mental health, indicated by low symptoms and high wellbeing, and the majority continue to do so as they enter early adolescence. However, results also indicated that a substantial proportion (36%) of children with initially good mental health deteriorated over that period. The most common transition was to a *vulnerable* or *emotional symptoms but content* status. Other longitudinal dual-factor studies have indicated that those who transition from *complete mental health* are most likely to move to a *symptomatic but content* group (Compton, 2016; Kelly et al., 2012; McMahan, 2012). The present study indicated that, from childhood to early adolescence, the most common transition is specifically to an *emotional symptoms but content* group. Other studies have also indicated that the transition from *complete mental health* to *vulnerable* is common in adolescence (Xiong et al., 2017; Zhou et al., 2020) and this is in line with the current findings. Together results suggest that interventions that aim to prevent mental health difficulties developing as children move into early adolescence should particularly focus on preventing emotional difficulties and increasing wellbeing, as those aspects of mental health are most likely to deteriorate.

The *vulnerable* status was the least stable over time and *vulnerable* youth were most likely to transition to a *complete mental health* status. Other studies have found that the *vulnerable* group was the least stable among younger (Compton, 2016) and older age groups (Kelly et al., 2012). However, the *vulnerable* status seems to be more stable among Chinese adolescents (Xiong et al., 2017; Zhou et al., 2020) suggesting that in some cultures a *vulnerable* status may be less of a transient experience, but further research is needed to verify that. However, it has been consistently indicated that those with a *vulnerable* status tend to transition to *complete mental health* status, indicating recovery (Compton, 2016; Kelly et al., 2012; McMahan et al., 2012; Xiong et al., 2017). Despite some suggestion that low wellbeing may lead to later mental health difficulties (Huppert, 2009), findings from the present study suggest that low subjective wellbeing is a transient experience for many children, which often improves over time. That said, it is notable that a quarter of those in the *vulnerable* group in childhood changed to a symptomatic status by early adolescence. Further investigation into what distinguishes those that make positive and negative transitions is warranted so that appropriate support can be offered.

Other noteworthy findings were that children with *conduct problems but content* or *troubled* status were most likely to remain in one of the symptomatic groups during early adolescence. This indicates the persistence of conduct problems and co-morbid symptoms, which has been demonstrated in other studies (e.g., Basten et al., 2016). Children experiencing those symptoms may need more intensive interventions to help them through the transition to early adolescence. In contrast, those with an *emotional symptoms but content* status in childhood had a similar chance of moving to a non-symptomatic group (i.e., *complete mental health* or *vulnerable*) as remaining in a symptomatic group (*emotional symptoms but content*, *conduct problems but content*, or *troubled*), suggesting that positive and neutral/negative changes are equally likely for children with emotional symptoms. That raises questions regarding the factors that influence those different developmental patterns, of which some were investigated in the current study.

### Sex Differences and Dual-Factor Mental Health

A secondary aim of the study was to investigate factors associated with changes in dual-factor mental health. The results indicated that, in childhood, the key sex differences were in terms of the types of symptoms experienced. As predicted, males were more likely to have a *conduct problems but content* status and females were more likely to have an *emotional symptoms but content* status. Furthermore, of those that had *complete mental health* in childhood, more males transitioned to the *conduct problems but content* group, and more females transitioned to the *emotional symptoms but content* group in early adolescence. This supports previous findings that indicate differences in how males and females experience and express distress (Merikangas et al., 2009) and shows how sex differences in childhood continue to develop into early adolescence. Findings align with previous research demonstrating that females tend to show increased emotional difficulties as they enter adolescence (Patalay & Gage, 2019).

Beyond differences in symptom dimensionality, associations between sex and specific developmental pathways during the passage to early adolescence were also identified. Females with *complete mental health* in childhood were more likely to move to a *vulnerable* status in early adolescence compared to males with *complete mental health*. It was also found that females with a *vulnerable* mental health status in childhood were at greater risk of developing emotional symptoms in early adolescence. Although the majority of children with *vulnerable* status in childhood recovered by early adolescence, those that were *vulnerable and female* were at greater risk of moving to an *emotional symptoms but content* status. This implies that mental health interventions could be targeted at this group to prevent the onset of emotional symptoms. Future research should also investigate why females are most at risk of following those specific pathways of mental health. One possibility is that hormonal changes are at least partly responsible. Research suggests that the onset of puberty can lead to an increase in emotional symptoms (Alloy et al., 2016) and females typically start puberty before males. Future work in this area may include the onset of puberty as a covariate. Overall, findings indicate that there are nuanced sex differences that emerge during the developmental period from childhood to early adolescence.

### Perceived Peer Social Support and Dual-Factor Mental Health Status and Transitions

The study findings also indicated the importance of perceived peer support in mental health development from childhood to early adolescence. As predicted, low peer support was associated with having a suboptimal mental health status (specifically *vulnerable* or *troubled*) in childhood, rather than having *complete mental health*. This supports previous variable-oriented research that has consistently found that peer support is positively associated with subjective wellbeing and negatively associated with mental health symptoms (Chu et al., 2010; Rueger et al., 2016). Further, findings showed that low peer support at age 8–9 years old was associated with specific mental health transitions as they entered early adolescence. Lower levels of peer support were associated with transitions from *complete mental health* in childhood to the *vulnerable* group in early adolescence. Further, low peer support was associated with maintenance of *emotional symptoms but content* status from childhood to early adolescence, rather than recovery into *complete mental health* status, and associated with transitions from *emotional symptoms but content* to the *troubled* status. In other words, low peer support was associated with specific patterns of deteriorating mental health (e.g., moving from *complete mental health* to *vulnerable*) rather than all negative mental health transitions.

The study showed that low peer support is an important risk factor for mental health during the period from childhood and adolescence. Missing from the analyses is the impact of other types of social support, such as caregiver or teacher support, during this transitional period, and future work will want to explore how support within families or from school influences the stability of mental health profiles, but also how they work with peer support, and sex, to predict changing profiles of mental health and well-being. For example, research has shown that females report receiving the most support from close friends, whilst males report receiving the most support from parents and teachers (Rueger et al., 2010). And, while a meta-analysis found that there are more sex similarities than differences, with both peer and family support having a moderate protective effect against depressive symptoms for both males and females (Rueger et al., 2016), such examinations should be conducted for work taking a dual-factor approach to understanding mental health and wellbeing. Further, results indicate that a lack of peer support is linked to poorer mental health, but it is not known what effect negative relationships (e.g., bullying) have. Bullying has been consistently linked to poorer mental health among children and adolescents (Arseneault, 2018) but it will be important to investigate the precise nature of mental health changes when mental health symptoms and wellbeing are simultaneously considered, particularly during the developmental period from childhood to early adolescence, because this is a time when sensitivity to the social environment increases and therefore the damaging effects of bullying may be the greatest (Sebastian et al., 2010).

### Implications for Child and Adolescent Mental Health Practice

The findings are important for understanding mental health development through the transitional period from childhood to early adolescence. They also have implications for child and adolescent mental health practice, particularly in community settings, such as schools, which are interested in assessing mental health in general population samples, identifying any issues early, and providing appropriate early intervention. First, results emphasize the importance of assessing different dimensions of symptoms (internalizing and externalizing) as well as subjective wellbeing, when undertaking mental health screening. Focusing on a single element would fail to identify the different dual-factor and symptom-specific groups that were empirically defined in this study, and which were shown to have distinct characteristics and developmental pathways. Identifying individuals with particular patterns of dual-factor mental health and dominant symptom type, allows identification of those that are already symptomatic *and* those at risk of worsening mental health. Therefore, interventions can be selected to address the specific needs of the child. For example, children in the *emotional symptoms but content* or *conduct problems but content* subgroup would benefit from interventions that help manage their specific symptoms in addition to any underlying issues that are causing their distress. Those in the *troubled* subgroup may require more intensive indicated interventions, and those in the *vulnerable* subgroup may benefit more from more preventative or promotive interventions that aim to improve wellbeing and resilience. Further, given that low peer support was associated with specific patterns of deteriorating mental health, interventions focused on social skills development and building close relationships might be appropriate for specific groups of young people to mitigate against declining mental health.

A second implication is that any dual-factor mental health screening, such as might be carried out in universal school-based screening programs (e.g., Dowdy et al., 2015), is carried out frequently given that dual-factor mental health is changeable. Over this two-year period, approximately half of the children experienced qualitative changes in their mental health status. Further research is required to establish the stability of dual-factor mental health over shorter and longer periods; however, a single round of screening during primary/elementary school may not adequately reflect mental health in early adolescence.

Third, this study has begun to characterize young people that make specific mental health transitions, with implications for identifying those at risk of deteriorating mental health. For example, the results suggested that those with a *complete mental health* status in childhood who are female and/or have low peer support, are more likely to move to the *vulnerable* status than remain in the complete mental health status in early adolescence; children with *emotional symptoms but content* status and low peer support are more likely to maintain that status or deteriorate further into the *troubled* status in early adolescence. This characterization can help to identify those most in need of support and future research should extend this to include a wider range of sociodemographic and psychosocial factors that have been associated with mental health such as socioeconomic status (Reiss et al., 2019), support from other sources (Rueger et al., 2010), and bullying (Arseneault, 2018). In addition, findings suggest that focusing on improving peer support may be more useful for some subgroups than others. A number of existing school-based interventions aim to improve children’s social skills and relationships and there is evidence to suggest that they help to improve children’s mental health and wellbeing (Durlak et al., 2011). The findings from the current study suggest larger effects may be seen for particular groups, such as those with *emotional symptoms but content* as levels of social support appeared to be linked to recovery or deterioration, although this would need further investigation. In addition, the characteristics of those showing different patterns of deteriorating or improving mental health could be investigated in greater depth through the use of additional covariates.

### Strengths, Limitations, and Future Directions

The study had many strengths including a longitudinal design, a relatively large sample, use of model-based classification methods (LTA), and an additional focus on factors associated with dual-factor mental health development over time. However, there are several limitations and directions for future research that warrant discussion. First, replication of the LTA in additional samples is necessary to indicate the extent to which the findings are reliable. Replicating this in larger samples would also be advantageous. Although the sample was large enough to identify classes and carry out LTA, when investigating covariate effects on transitions, the number of children making some of the transitions was very low, therefore the analysis may not have been sufficiently powered to detect covariate relationships. Further, in the current study we used a general population sample that included those with special educational needs and disabilities (SEND). Children with SEND may be more likely to have mental health difficulties (McMillan & Jarvis, 2013) and inclusion of those with SEND in the current study may have influenced the mental health classes that were found. However, the current study was interested in mental health among general population samples, of which a proportion would be expected to have SEND, therefore, excluding students on that basis was not deemed appropriate. Future research may wish to investigate dual-factor mental health development for those with and without SEND to better understand any differences.

Second, there were some limitations in terms of the measurement of variables. For example, the assessment of subjective wellbeing was limited in that it focused mainly on positive affect. Some researchers would argue that life-satisfaction is an important component of subjective wellbeing (e.g., Diener, 2000) however studies have indicated that positive affect is an adequate proxy (Huta, 2015). Further, it is noted that by simplifying the response scales for mental health items to reduce model complexity for the LTA model, detailed information on symptom severity and extent of subjective wellbeing has been lost. As computational processing capacity increases, future studies may be able to address this. It is also noted that wellbeing items and symptom items were taken from different scales. Emotional symptoms and conduct problems were measured using the teacher report version of the Strengths and Difficulties Questionnaire. This is consistent with other studies that have investigated dual-factor mental health among children (e.g., Smith et al., 2020), reflecting that many measures of child mental health are not available in self-report versions. However, subjective wellbeing was measured using a self-report measure (KIDSCREEN-27), as was perceived peer support. Conceptually this is appropriate as both are subjective, however, this may have led to increased associations between social support and those mental health classes distinguished by wellbeing. Ideally the same type of measure and informant would be used to assess both symptoms and wellbeing. Unfortunately, mental health surveys tend to focus on either psychopathology or wellbeing, therefore, dual-factor studies need to combine scales from different surveys; furthermore, secondary analyses, such as that presented in the current study, are required to work with the data that are available, which in this case meant that using data from more than one informant. Methodological research is needed to assess how unharmonized measures effect findings in these types of analyses. In addition, the psychometric validity of the items included in the LCA is not clear because psychometric testing is typically completed at the scale level rather than the item level. Psychometric studies that look at the validity and reliability of single items is needed. Finally, in terms of social support, we looked only at peer social support, and future work will want to explore how support from others (such as caregivers and teachers) and damaging relationships such as bullying, work independently and interact with peer support to predict dual-factor statuses.

Finally, it is recognized that observed effect sizes were small in terms of the relationship between social support and mental health classes. Confidence intervals for odds ratios were close to 1 in many cases. Mental health development is complex with many contributing factors so no one factor would be expected to have large associations with mental health (Ungar & Theron, 2020). However, small effects could become important if they amplified over time (Anvari et al., 2021), which suggest the importance of further investigating potential cascading effects and interactions with other risk and resilience factors.

## Conclusion

The dual-factor approach recognizes that mental health is complex, comprising of both symptoms and wellbeing. Although there is increasing support for this model of mental health, little is known about how dual-factor mental health changes over time, particularly during key developmental periods, or which factors predict change. The current study investigated dual-factor mental health during the important developmental period from childhood to early adolescence and investigated whether sex or perceived support from peers was associated with specific changes in mental health. Findings indicated that changes in dual-factor mental health during this time were common and, while population level research suggests that mental health tends to deteriorate from childhood to early adolescence, the current findings show that this only applies to a particular subset of the population. *Complete mental health* was the most common and stable status. However, many young people showed deterioration in mental health during the study period, and sex and peer support were associated with some of those changes. For example, females and those with low peer support were more likely to move from a *complete mental health* to a *vulnerable* status; females were more likely to move to statuses characterized by emotional problems, whereas males moved to statuses characterized by behavior problems; and low peer support was associated with the maintenance of an *emotional symptoms but content* status and transitions from *emotional symptoms but content* to *a troubled* status. The findings demonstrate heterogeneous developmental pathways and flux with regards to dual-factor mental health development during the transition from childhood to early adolescence, and the current study has begun to characterize children who follow specific changes in dual-factor mental health. The findings have implications for how we assess mental health among children and young people as considering mental health symptom *and* subjective wellbeing identifies distinct subgroups that have different developmental trajectories and characteristics. Moving forward, there are opportunities to investigate the replicability of these results among other groups of adolescents and over different time periods. It will also be important to investigate other predictors of mental health transitions, such as sociodemographic variables, other forms of support (e.g., from carers or teachers) and the impact of damaging relationships (e.g., bullying) in order to better characterize those important groups, and to develop strategies to improve child and adolescent mental health during this period.

## Supplementary Information

Online Resource 1

Online Resource 2

Online Resource 3

Online Resource 4

Online Resource 5

Online Resource 6

Online Resource 7

## Data Availability

The manuscript’s data will not be deposited.

## Footnotes

1 77 schools were involved in the original trial, but one school failed to provide mental health data for students in the final phase and, therefore, was not included in the current study.

2 A fifth item “often steals” was removed due to low endorsement of the item. It was endorsed for 2.5% and 3.1% of students age 8–9 years and 10–11 years respectively.

3 Ethnicity was not included as a covariate in the current study due to concerns around statistical power (i.e., even with a relatively large sample, the N for some ethnic groups was very small and the resulting coefficient values would likely be spurious).

4 Results indicated that the intervention trial arm was not significantly associated with mental health status or mental health transitions.

5 The model that used list-wise deletion of cases with missing data on the x variables has been included as a sensitivity analysis (see Online Resources 6 and 7). In both analyses the overall pattern of results remained the same.

6 Full covariate results are presented in Online Resources 4 and 5. Sensitivity analyses are also presented in Online Resources 6 and 7. Note that the same pattern of covariate results was found in the sensitivity analysis as presented here.
