# Supplementary material for: Dual-Factor Mental Health from Childhood to Early Adolescence and Associated Factors: A Latent Transition Analysis
Source: J Youth Adolesc. 2021 Dec 17;51(6):1118–33. doi: 10.1007/s10964-021-01550-9 (PMC9090675; doi:10.1007/s10964-021-01550-9)
Supplement: Supplementary file 1 — Online Resource 1 [file 10964_2021_1550_MOESM1_ESM.docx]

| **Online Resource 1**  *Average Responses on Mental Health and Peer Support Variables*  *When Age 8-9 Years (T1) and 10-11 Years (T2)* | | | | | |
| --- | --- | --- | --- | --- | --- |
|  | T1 | | T2 | |  |
| Endorsement of mental health symptoms | Freq. | % | Freq. | % |  |
| CP1 temper | 300 | 13 | 356 | 15 |  |
| CP2 obedient R | 547 | 23 | 579 | 24 |  |
| CP3 fights/bullies | 289 | 12 | 320 | 13 |  |
| CP4 lies/cheats | 317 | 13 | 300 | 13 |  |
| ES1 headaches | 372 | 16 | 352 | 15 |  |
| ES2 worries | 581 | 24 | 650 | 27 |  |
| ES3 unhappy | 392 | 16 | 437 | 18 |  |
| ES4 nervous | 432 | 18 | 503 | 21 |  |
| ES5 fear | 317 | 13 | 396 | 17 |  |
| Mean scores for subjective wellbeing items | M | SD | M | SD |  |
| SWB1 life enjoyable | 3.83 | 1.12 | 3.92 | 0.93 |  |
| SWB2 good mood | 3.74 | 1.01 | 3.69 | 0.90 |  |
| SWB3 had fun | 4.16 | 1.02 | 3.98 | 0.89 |  |
| SWB4 happy self | 4.23 | 1.14 | 4.15 | 1.10 |  |
| Mean score on peer support scale | M | SD | M | SD |  |
| peer support | 51.62 | 11.54 | 51.91 | 10.23 |  |
| *Note.* T1 = Time 1 (age 8-9 years); T2 = Time 2 (age 10-11 years); CP = conduct problems; ES =emotional symptoms; SWB = subjective wellbeing.  SWB items are on a scale of 1-5. | | | | |  |
