# Supplementary material for: Dual-Factor Mental Health from Childhood to Early Adolescence and Associated Factors: A Latent Transition Analysis
Source: J Youth Adolesc. 2021 Dec 17;51(6):1118–33. doi: 10.1007/s10964-021-01550-9 (PMC9090675; doi:10.1007/s10964-021-01550-9)
Supplement: Supplementary file 2 — Online Resource 2 [file 10964_2021_1550_MOESM2_ESM.docx]

**Online Resource 2**

*Plots Showing the Results of the 4- and 5-Class LCA Models at Both Time Points.*

*(a) probability of endorsing symptoms and (b) mean scores on subjective wellbeing items (prevalence included in the legend)*

1) T1 (age 8-9) 4-class model

**
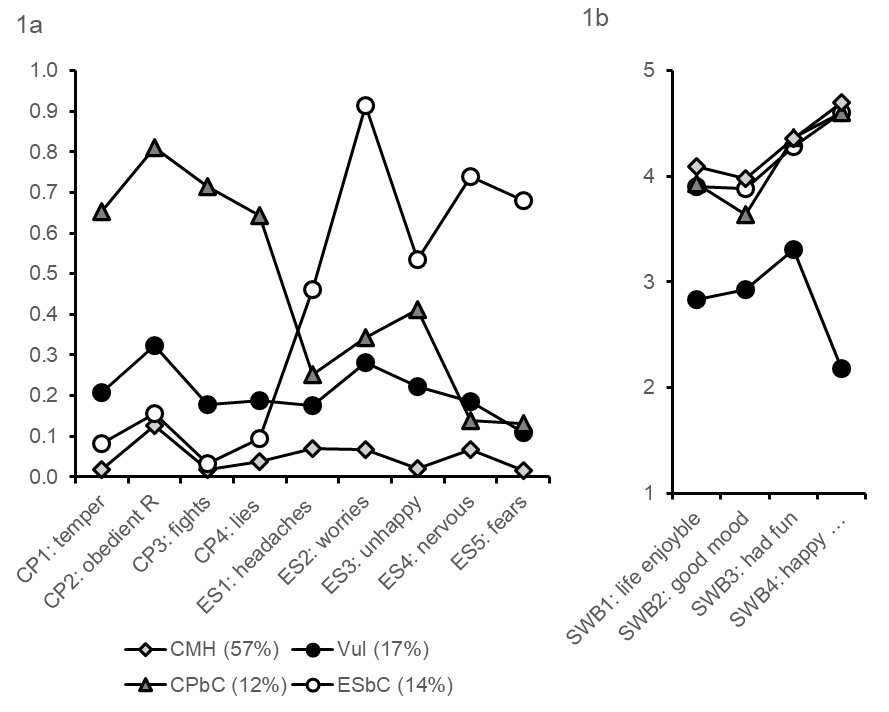
**

2) T2 (age 10-11) 4-class model

**
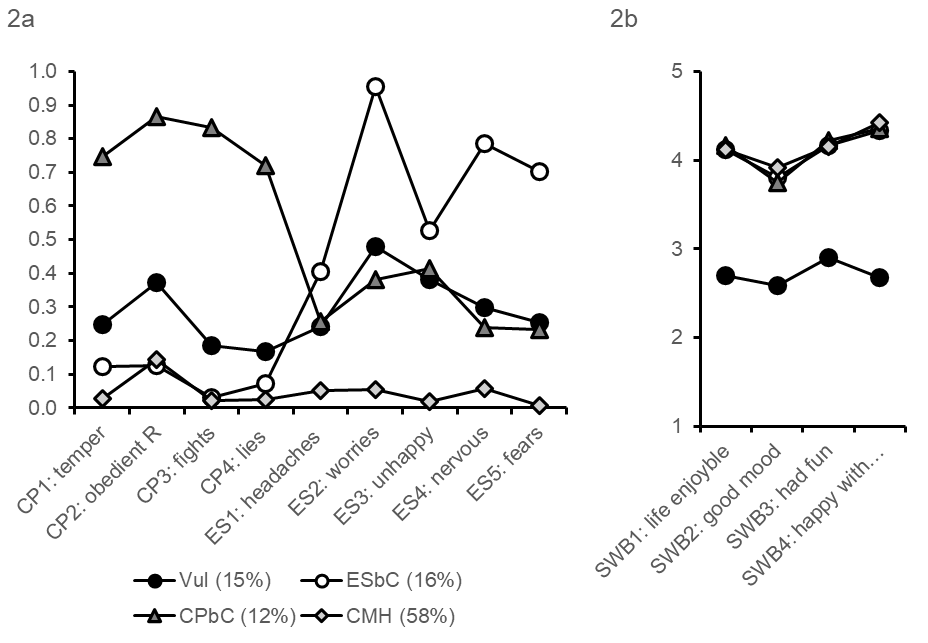
**

*Online Resource 2 (continued)*

3) T1 (age 8-9) 5-class model


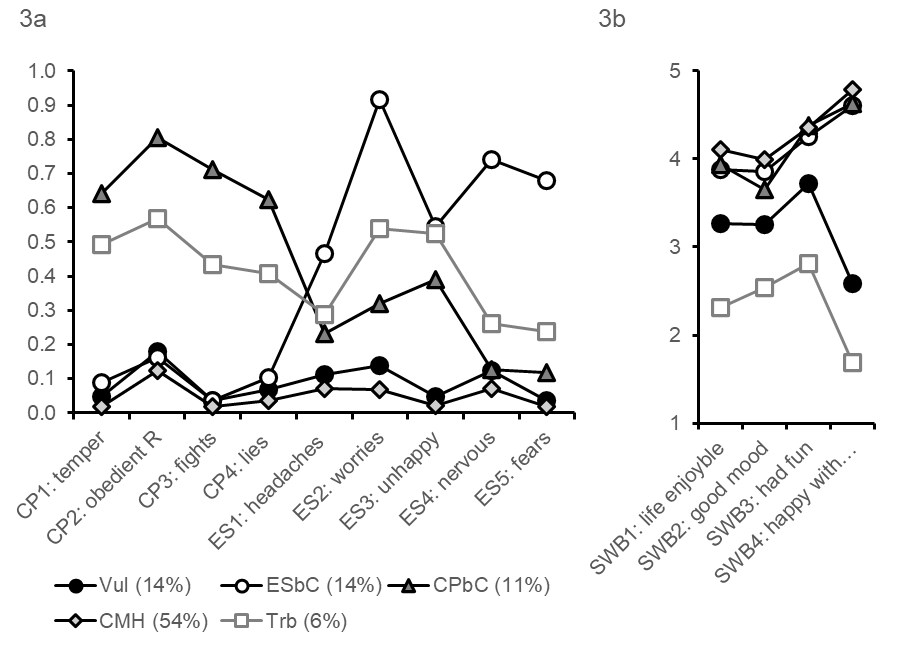


4) T12 (age 10-11) 5-class model

*
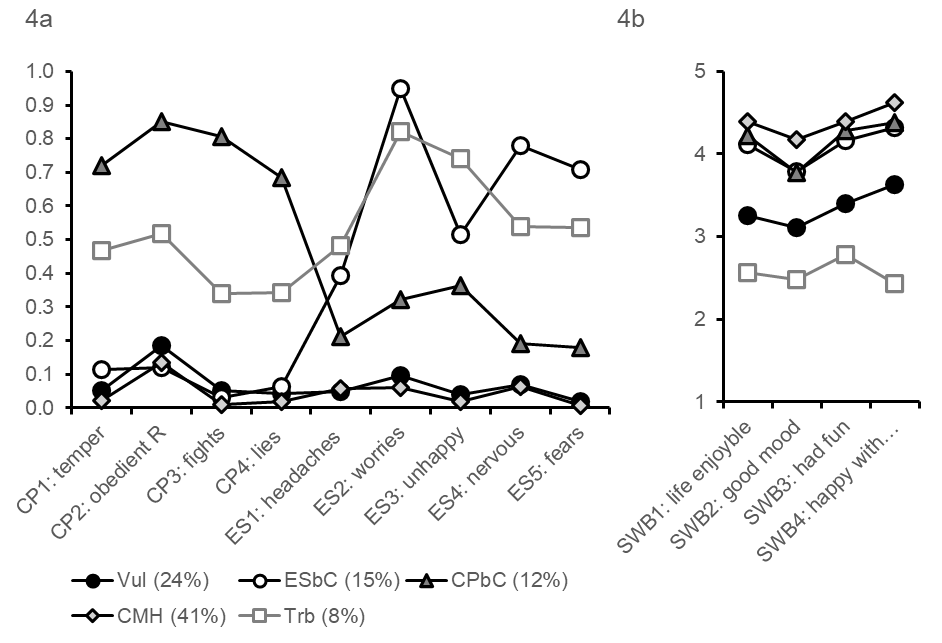
*

*Note.* CP = conduct problems; ES = emotional symptoms; SWB = subjective wellbeing;

CMH= complete mental health; Vul = vulnerable; ESbC = emotional symptoms but content;

CPbC = conduct problems but content; Trb = troubled.
