# Supplementary material for: Dual-Factor Mental Health from Childhood to Early Adolescence and Associated Factors: A Latent Transition Analysis
Source: J Youth Adolesc. 2021 Dec 17;51(6):1118–33. doi: 10.1007/s10964-021-01550-9 (PMC9090675; doi:10.1007/s10964-021-01550-9)
Supplement: Supplementary file 3 — Online Resource 3 [file 10964_2021_1550_MOESM3_ESM.docx]

| **Online Resource 3**  *Longitudinal Invariance Testing: Information Criteria for Models Indicating Configural, Structural, Dispersion, and Distributional Similarity* | | | | |
| --- | --- | --- | --- | --- |
| Model | BIC | ssaBIC | AIC | CAIC |
| Configural similarity^a^ | 78547.74 | 78083.86 | 77703.26 | 78050.83 |
| Structural similarity^b^ | 78412.74 | 78155.39 | 77944.23 | 78137.06 |
| Dispersion similarity^c^ | 78571.81 | 78327.17 | 78126.44 | 78309.75 |
| Distributional similarity^d^ | 78548.17 | 78316.23 | 78125.93 | 78299.71 |
| *Note.* BIC = Bayesian Information Criterion; ssaBIC = sample size adjusted BIC; AIC = Akaike’s Information Criteria; CAIC = Consistent AIC.  ^a^ unrestricted 5-class model;  ^b^ item response probabilities and held equivalent over time.  ^c^ item response probabilities, means, and variances held equivalent over time.  ^d^ item response probabilities, means, variances, and class prevalence’s held equivalent over time. | | | | |
