# Supplementary material for: Dual-Factor Mental Health from Childhood to Early Adolescence and Associated Factors: A Latent Transition Analysis
Source: J Youth Adolesc. 2021 Dec 17;51(6):1118–33. doi: 10.1007/s10964-021-01550-9 (PMC9090675; doi:10.1007/s10964-021-01550-9)
Supplement: Supplementary file 5 — Online Resource 5 [file 10964_2021_1550_MOESM5_ESM.docx]

| **Online Resource 5**  *Covariate Results for Mental Health Transitions* | | | | | | |
| --- | --- | --- | --- | --- | --- | --- |
| T1 mental health status | T2 mental health status* | Covariate | OR | OR 95% CI | |  |
|  |  |  |  | LL | UL |  |
| Complete mental health | Vulnerable | **Male** | **0.53** | **0.32** | **0.87** |  |
|  |  | **Peer support** | **0.98** | **0.96** | **0.99** |  |
|  | Emotional symptoms but content | **Male** | **0.40** | **0.25** | **0.65** |  |
|  |  | Peer support | 1.00 | 0.97 | 1.02 |  |
|  | Conduct problems but content | **Male** | **2.80** | **1.36** | **5.76** |  |
|  |  | Peer support | 1.01 | 0.97 | 1.05 |  |
|  | Troubled | Male | 0.75 | 0.36 | 1.57 |  |
|  |  | Peer support | 0.97 | 0.93 | 1.02 |  |
| Vulnerable | Vulnerable | Male | 0.46 | 0.21 | 1.02 |  |
|  |  | Peer support | 0.99 | 0.94 | 1.00 |  |
|  | Emotional symptoms but content | **Male** | **0.23** | **0.07** | **0.73** |  |
|  |  | Peer support | 0.98 | 0.94 | 1.02 |  |
|  | Conduct problems but content | Male | 13.59 | 0.01 | 29269.82 |  |
|  |  | Peer support | 1.02 | 0.95 | 1.10 |  |
|  | Troubled | Male | 0.30 | 0.09 | 1.01 |  |
|  |  | Peer support | 0.99 | 0.89 | 1.10 |  |

*Online Resource 5 (continued)*

| T1 mental health status | T2 mental health status* | Covariate | OR | OR 95% CI | |
| --- | --- | --- | --- | --- | --- |
|  |  |  |  | LL | UL |
| Emotional symptoms but content | Vulnerable | Male | 0.88 | 0.30 | 2.55 |
|  |  | Peer support | 0.96 | 0.91 | 1.01 |
|  | Emotional symptoms but content | Male | 0.88 | 0.39 | 1.97 |
|  |  | **Peer support** | **0.95** | **0.91** | **0.99** |
|  | Conduct problems but content | Male | 1.46 | 0.09 | 24.36 |
|  |  | Peer support | 0.96 | 0.80 | 1.16 |
|  | Troubled | Male | 1.93 | 0.65 | 5.77 |
|  |  | **Peer support** | **0.94** | **0.88** | **0.99** |
| Conduct problems but content | Vulnerable | Male | 0.93 | 0.13 | 6.58 |
|  |  | Peer support | 0.99 | 0.92 | 1.08 |
|  | Emotional symptoms but content | Male | 2.29 | 0.20 | 26.19 |
|  |  | Peer support | 1.01 | 0.92 | 1.11 |
|  | Conduct problems but content | Male | 2.99 | 0.95 | 9.41 |
|  |  | Peer support | 1.01 | 0.97 | 1.06 |
|  | Troubled | Male | 0.46 | 0.10 | 2.07 |
|  |  | Peer support | 0.96 | 0.89 | 1.04 |
| Troubled | Vulnerable | Male | 1.12 | 0.16 | 7.88 |
|  |  | Peer support | 1.00 | 0.92 | 1.08 |
|  | Emotional symptoms but content | Male | 0.96 | 0.14 | 6.69 |
|  |  | Peer support | 1.03 | 0.96 | 1.09 |
|  | Conduct problems but content | Male | 3.24 | 0.34 | 31.29 |
|  |  | Peer support | 1.03 | 0.94 | 1.11 |
|  | Troubled | Male | 0.91 | 0.19 | 4.45 |
|  |  | Peer support | 1.00 | 0.94 | 1.08 |
| *Note.* Bolded values are statistically significant, i.e., 95% odds ratios do not cross 1.  T1 = Time 1 (age 8-9 years); T2 = Time 2 (age 10-11 years); OR = odds ratio.  *Complete mental health as reference class | | | | | |
