# Supplementary material for: Dual-Factor Mental Health from Childhood to Early Adolescence and Associated Factors: A Latent Transition Analysis
Source: J Youth Adolesc. 2021 Dec 17;51(6):1118–33. doi: 10.1007/s10964-021-01550-9 (PMC9090675; doi:10.1007/s10964-021-01550-9)
Supplement: Supplementary file 6 — Online Resource 6 [file 10964_2021_1550_MOESM6_ESM.docx]

| **Online Resource 6**  *Sensitivity Analyses: Covariate Results for Mental Health Statuses at T1 (8-9 years) when Missing Data on Covariates is Deleted List-Wise (N= 2240)* | | | | | |  |
| --- | --- | --- | --- | --- | --- | --- |
| T1 mental health status* | Covariate | OR | OR 95% CI | |  |  |
|  |  |  | LL | UL |  | |
| Vulnerable | Male | 1.01 | 0.79 | 1.30 |  | |
|  | **Peer support** | **0.94** | **0.92** | **0.96** |  | |
| Emotional symptoms but content | **Male** | **0.65** | **0.47** | **0.90** |  | |
|  | Peer support | 0.99 | 0.97 | 1.00 |  | |
| Conduct problems but content | **Male** | **2.51** | **1.71** | **3.70** |  | |
|  | Peer support | 1.00 | 0.98 | 1.02 |  | |
| Troubled | Male | 1.48 | 0.91 | 2.42 |  | |
|  | **Peer support** | **0.91** | **0.89** | **0.93** |  | |
| *Note.* Bolded values are statistically significant, i.e., 95% odds ratios do not cross 1.  T1 = Time 1 (age 8-9 years); OR = odds ratio.  *Complete mental health as reference class | | | | |  |  |
