# Supplementary material for: Dual-Factor Mental Health from Childhood to Early Adolescence and Associated Factors: A Latent Transition Analysis
Source: J Youth Adolesc. 2021 Dec 17;51(6):1118–33. doi: 10.1007/s10964-021-01550-9 (PMC9090675; doi:10.1007/s10964-021-01550-9)
Supplement: Supplementary file 7 — Online Resource 7 [file 10964_2021_1550_MOESM7_ESM.docx]

| **Online Resource 7**  *Sensitivity Analysis: Covariate Results for Mental Health Transitions when Missing Data on Covariates is Deleted List-Wise (N= 2240)* | | | | | | |
| --- | --- | --- | --- | --- | --- | --- |
| T1 mental health status | T2 mental health status* | Covariate | OR | OR 95% CI | |  |
|  |  |  |  | LL | UL |  |
| Complete mental health | Vulnerable | **Male** | **0.51** | **0.30** | **0.86** |  |
|  |  | **Peer support** | **0.98** | **0.96** | **0.99** |  |
|  | Emotional symptoms but content | **Male** | **0.38** | **0.23** | **0.64** |  |
|  |  | Peer support | 0.99 | 0.97 | 1.03 |  |
|  | Conduct problems but content | **Male** | **2.92** | **1.39** | **6.13** |  |
|  |  | Peer support | 1.01 | 0.97 | 1.05 |  |
|  | Troubled | Male | 0.92 | 0.45 | 1.92 |  |
|  |  | Peer support | 0.97 | 0.93 | 1.02 |  |
| Vulnerable | Vulnerable | Male | 1.46 | 0.19 | 1.01 |  |
|  |  | Peer support | 1.46 | 0.94 | 1.00 |  |
|  | Emotional symptoms but content | **Male** | **0.26** | **0.08** | **0.82** |  |
|  |  | Peer support | 0.98 | 0.94 | 1.02 |  |
|  | Conduct problems but content | Male | 12.99 | 0.01 | 21087.63 |  |
|  |  | Peer support | 1.02 | 0.95 | 1.09 |  |
|  | Troubled | Male | 0.31 | 0.10 | 1.01 |  |
|  |  | Peer support | 0.99 | 0.90 | 1.10 |  |

*Online Resource 7 (continued)*

| T1 mental health status | T2 mental health status* | Covariate | OR | OR 95% CI | |
| --- | --- | --- | --- | --- | --- |
|  |  |  |  | LL | UL |
| Emotional symptoms but content | Vulnerable | Male | 1.08 | 0.36 | 3.29 |
|  |  | Peer support | 0.95 | 0.90 | 1.01 |
|  | Emotional symptoms but content | Male | 0.97 | 0.41 | 2.26 |
|  |  | **Peer support** | **0.95** | **0.91** | **0.99** |
|  | Conduct problems but content | Male | 1.44 | 0.09 | 23.04 |
|  |  | Peer support | 0.95 | 0.81 | 1.13 |
|  | Troubled | Male | 2.44 | 0.78 | 7.60 |
|  |  | **Peer support** | **0.94** | **0.88** | **0.99** |
| Conduct problems but content | Vulnerable | Male | 0.94 | 0.13 | 7.07 |
|  |  | Peer support | 1.00 | 0.92 | 1.07 |
|  | Emotional symptoms but content | Male | 3.14 | 0.25 | 40.14 |
|  |  | Peer support | 1.01 | 0.92 | 1.11 |
|  | Conduct problems but content | Male | 2.76 | 0.83 | 9.20 |
|  |  | Peer support | 1.02 | 0.97 | 1.06 |
|  | Troubled | Male | 0.42 | 0.08 | 2.13 |
|  |  | Peer support | 0.96 | 0.88 | 1.04 |

*Online Resource 7 (continued)*

| T1 mental health status | T2 mental health status* | Covariate | OR | OR 95% CI | |
| --- | --- | --- | --- | --- | --- |
|  |  |  |  | LL | UL |
| Troubled | Vulnerable | Male | 0.89 | 0.12 | 6.59 |
|  |  | Peer support | 0.99 | 0.92 | 1.07 |
|  | Emotional symptoms but content | Male | 0.10 | 0.70 | 4.95 |
|  |  | Peer support | 1.03 | 0.96 | 1.09 |
|  | Conduct problems but content | Male | 2.85 | 0.29 | 28.45 |
|  |  | Peer support | 1.02 | 0.94 | 1.11 |
|  | Troubled | Male | 0.81 | 0.15 | 4.32 |
|  |  | Peer support | 1.00 | 0.93 | 1.07 |
| *Note.* Bolded values are statistically significant, i.e., 95% odds ratios do not cross 1;  T1 = Time 1 (age 8-9 years); T2 = Time 2 (age 10-11 years); OR = odds ratio.  *Complete mental health as reference class | | | | | |
